# Artificial intelligence to improve patient care in emergency medicine: a workflow-based analysis

**DOI:** 10.1007/s11739-025-04155-3

**Published:** 2025-11-14

**Authors:** Francesco Franceschi, Prabakar Vaittinada Ayar, Taj Hassan, André Gries

**Affiliations:** 1 Emergency Department, Fondazione Policlinico Universitario A. Gemelli IRCCS, Università Cattolica del Sacro Cuore, Rome, Italy; 2 Emergency Department, University Hospital of Orléans, University of Orléans, Orleans, France; 3 Centre for Emergency Care & Global Health, Dept of Emergency Medicine, Leeds Teaching Hospitals, Leeds, UK; 4 Emergency Department, Observation Unit, University Hospital of Leipzig, Leipzig, Germany

**Keywords:** Artificial intelligence, Emergency Medicine, In-hospital, Pre-hospital

## Abstract

In the last years, artificial intelligence has had a strong impact on health sciences, including emergency medicine. There are different fields of application, from pre-hospital to in-hospital issues. Concerning pre-hospital care, it may be useful in controlling patient’ transportation by public ambulance in emergency departments and improve transport time outliers. In hospital management may benefit from its ability to read out imaging or to rapidly calculate predictive scores or suggest therapeutic strategies. While the application of artificial intelligence in emergency medicine is surely intriguing, it is not free from potential risks, which in turn may overcome benefits. Since the majority of the studies are very small rather than pilot, a clear discussion among EM physicians is now necessary in order to better define the application of this technology in the real world by maximizing benefits and reducing risks.

In the last years, Artificial Intelligence (AI) has had a strong impact in the field of medicine, including emergency medicine (EM). Indeed, the hypothesis that some typically challenging medical activities may be improved by AI represents a major step forward both in terms of impact on patient care, as well as improving the working conditions of healthcare professionals. This is why many authors in the last years explored the possibility to apply AI in improving patient care in the emergency setting [1]. Just to give an idea of the impact of AI in the field, there are currently 154 AI-enabled products available for EM, most of them applicable for emergency imaging [2], while the Canadian Association of Emergency Physicians Research Symposium, in order to summarize the most relevant topics in which AI may be applied, has identified 11 specific recommendations for AI in EM [3].

This paper represents a viewpoint on how AI may improve patient care across each phase of emergency care based on the available literature and with a look to the future. The key areas will be to improve efficiency and effectiveness of care delivery and augmenting the decision-making process of clinicians (Fig. 1). The ultimate indicator of success will be improved patient outcome and impacting positively on premature ‘clinician burnout’.

**Fig. 1:**
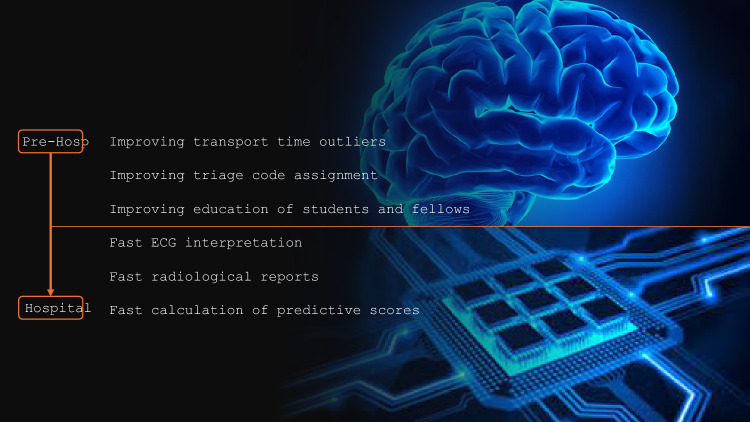
Possible applications of artificial intelligence in Emergency Medicine, from pre-hospital to hospital care

It is known that one of the main problems of pre-hospital emergency systems is where to transport patients when different options are available. Kim and coworkers used a novel digital platform named CONNECT-AI (CONnected Network for EMS Comprehensive Technical-Support using Artificial Intelligence), able to collect real-time patient data from the scene and hospital resource information, such as bed occupancy and the availability of emergency surgeries or procedures, in order to test patient transfer delays in South Korea. A total of 14,853 patients transported by public ambulance were finally selected for analysis, with a median transport time of 10 (IQR 7–14) minutes in the intervention group and 9 (IQR 6–13) minutes in the control group. The application of CONNECT-AI system produced a significant reduction in outlier cases in patients with fever or respiratory symptoms (36.5%–30.1%, *P* = 0.01) as well as a lower mortality rate compared with the control group (1.54%–0.64%, *P* = 0.01). The authors have concluded that AI may be useful in controlling patient’ transportation by public ambulance in EDs, especially improving transport time outliers [4]. On the other hand, AI and machine-learning algorithm may support the monitoring of EDs crowding, thus giving the opportunity to modulate human resources inside EDs [5], as recently demonstrated by Akbasli et al., who extracted data from 352.843 pediatric ED admissions. Twenty time-series forecasting models, including classical methods and advanced deep learning architectures, such as Temporal Convolutional Network, Time-series Dense Encoder and Reversible Instance Normalization, Neural High-order Time Series model, and Neural Basis Expansion Analysis, were developed and compared using Python 3.8 software. Shift schedules were optimized based on forecasted patient volumes using integer linear programming. They demonstrated that advanced deep learning models worked better than traditional models, while this model increased physician allocation by up to 30.4% during peak hours. Moreover, this new model reduced the patient-to-physician ratio by an average of 4.32 patients during the 8–16 shift and 4.40 patients during the 16–24 shift. In the Policlinico Universitario A. Gemelli IRCCS of Rome, we have developed a similar model able to forecast patient flow in the ED based on day and month, very useful in order to modulate EM doctor shifts and also hospital beds necessary for in-patient admission from the ED.

Concerning Triage, AI-driven systems may be helpful in directing patients to opt for the most appropriate setting for their condition, possibly reducing inappropriate ED access. While this concept is very intriguing, current studies are very cautious since diverting patients from EDs to other settings could represent either a medical or a legal risk [6], as recently report by Zaboli et al., who retrospectively analyzed data from 2658 patients accessing the ED. Triage codes assigned by human triage personnel were compared with those assigned by Artificial Intelligence (AI) triage using Chat-GPT 4.0. ROC analysis demonstrated that human triage outperformed AI in many parameters, including 30-day mortality (0.88 vs 0.70; *P* < 0.001) and life-saving interventions (0.98 vs 0.87; *P* = 0.014). We have recently tested an AI-driven triage tool and verified that accuracy is questionable, since triage rules are different among different Countries and different regions and all systems need to be adjusted accordingly. Chatbots based on generative AI are the most used tools by EM doctors and students at the moment: They are also used for triage of patients. However, at this time the results have to be compared with standard triage by nurses.

Furthermore, to receive vital function for the triage automatically also AI supported devices could be used. In Germany, the system “SmED” is used by the “Kassenärztliche Vereinigung” to alocate patients to the GP, HEMS and the emergency department [7].

Post-triage care is the area where AI may play a major role, mostly due to the intrinsic characteristic of this technology. In fact, AI showed a satisfactory level in specific issues, such as data analysis, algorithm application and imaging. There are many studies exploring risk analysis for selected conditions, such as head trauma, sepsis, or cardiovascular diseases. Imaging analysis is also another topic in which AI may play a major role. Indeed, ECG interpretation has been the first issue in which AI has been applied, while machine learning techniques are fundamental in the management of patients with syncope or to predict the occurrence and outcome of cardiac arrest [8, 9]. Lee and coworkers, in particular, published the results of the Rule-Out acute Myocardial Infarction using Artificial intelligence Electrocardiogram analysis (ROMIAE) study, a prospective cohort study conducted in 18 Korean university-level teaching hospitals. Adult patients presenting to the ED within 24 h chest pain were assessed. Exposure included AI-ECG score, HEART score, GRACE 2.0 score, high-sensitivity troponin level, and physician AMI score. Primary outcome was diagnosis of AMI and the secondary outcome was 30 day major adverse cardiovascular event (MACE). 8493 adults, of whom 1586 (18.6%) were diagnosed with AMI, were enrolled; the integration of the AI-ECG improved risk stratification and AMI discrimination by 19.6% (95% CI, 17.38–21.89) and a C-index of 0.926 (95% CI, 0.919–0.933), compared with the HEART score alone [8]. Lu and coworkers designed a study aimed at addressing the early detection of Emergency Department in-hospital cardiac arrest (EDCA) by developing an innovative deep learning model, the ECG-Image-Aware Network (EIANet), which uses 12-lead ECG images. They enrolled 571 cases and 826 control, showing a significant improvement of EDCA prediction by 36%, while the feature map showed that the region of interest in the ECG was the ST segment [9].

Radiology is another important field of application of AI, where the use of selected algorithms allows to rapidly obtain reports from traditional radiology, POCUS or CT scan [10, 11]. Moreno and coworkers used an AI applied to urgent bone X-rays in order to detect fractures. They enrolled 792 patients accessing the ED and reported a 90.6% of sensitivity and 98% of specificity for fracture detection by radiologist compared to 95.8% of sensitivity and 97.6% of specificity by AI [10]. On the other hand, Baloescu et al. tested the ability of AI to guide the acquisition of diagnostic-quality lung ultrasound (LUS) images by trained health care professionals (THCPs). In this multicenter study, 176 participants with shortness of breath recruited from 4 clinical sites underwent two ultrasound examinations, one by a THCP operator using Lung Guidance AI and the other one by a trained LUS expert without AI. They demonstrated that the quality of LUS did not change, with a non-significant difference of only 1.7% between groups. This is a very important topic, since we know that the time of radiology exerts a very strong impact on ED length of stay (LOS) [11].

Artificial intelligence (AI) applications are increasingly recognized as valuable tools in the emergency department (ED) for the acute management of traumatic brain injury (TBI). AI-based image analysis enables rapid triage of head CT scans, with FDA-approved systems such as Aidoc demonstrating improved detection and timely notification of intracranial hemorrhage, thereby expediting radiological review and potentially reducing preventable mortality [12]. Beyond detection, advanced deep learning architectures such as the ASIST-TBI Vision Transformer have shown high accuracy (AUC ≈ 0.89) in predicting the need for urgent neurosurgical intervention directly from CT imaging, offering clinicians robust decision support in time-critical settings [13]. In parallel, supervised machine learning algorithms, including CatBoost, have been applied to clinical and imaging datasets to predict short-term mortality in high-risk populations, with promising discrimination (AUROC 0.867 in geriatric cohorts), thereby supporting triage, ICU transfer, and family counseling in the ED [14]. Collectively, these systems demonstrate that AI can complement clinical expertise by accelerating diagnostic workflows, guiding surgical decision-making, and improving risk stratification during the initial management of TBI in emergency care.

Finally, AI may be crucial in identifying critical biomarkers in emerging diseases, thus reducing medical errors through the underestimation of important lab parameters. Garrido et al. designed a study in which consecutively admitted patients with COVID-19 infection were analyzed for more than 89 variables using the Random Forest (RF) algorithm. The RF model showed that the biomarkers most predictive of mortality included procalcitonin (PCT), lactate dehydrogenase (LDH), and C-reactive protein (CRP). Moreover, this system highlighted the significance of interstitial infiltrates in chest X-rays and D-dimer levels. [15]. All those issues in the near future may surely improve post-triage activities, increasing the quality of care and reducing ED LOS.

AI is also an important tool to improve communication with patients or relatives accessing EDs from different countries. This may be crucial when healthcare professionals need to acquire important medical or non-medical information or obtain at least verbal consent for critical procedures. Concerning medical education, ChatGPT or other systems are often used among students to optimize study time, since AI is able to summarize book chapters or create virtual tests [16, 17]. However, Bahrami et al., reported that studies indicate that Google Translate and ChatGPT are not accurate enough for advanced medical terminology and we need to develop and implement an ad hoc machine translation tool for bridging language barriers [16]. Iftikhar and coworkers conducted a retrospective study in which EM residents’ examination scores were collected and compared with the performance of ChatGPT on the same examinations. They enrolled 238 residents and reported that ChatGPT scored consistently higher than resident groups in all examination categories, while a potential misalignment between examination performance and practical competencies was also detected [17]. The same systems may be also used to prepare doctors to design research protocols, run national board tests, or even write motivation letters. [18]. Finally, AI may be also used to overcome gender and ethnic appearance when healthcare professionals need to take a crucial decision. Gisselbaek et al. performed a study aimed at examining the demographic representation of two AI text-to-image models, Midjourney and ChatGPT DALL-E 2, and assessing their accuracy in depicting the demographic characteristics of intensivists. Interestingly, ChatGPT-DALL-E2 produced less female (17.3% vs 32.2%, *P* < 0.0001), more white (61% vs 55.1%, *P* = 0.002) and younger (53.3% vs 23.9%, *P* < 0.001) individuals, while Midjourney depicted more female (47.6% vs 32.2%, *P* < 0.001), more white (60.9% vs 55.1%, *P* = 0.003) and younger intensivist (49.3% vs 23.9%, *P* < 0.001) [19]. All those issues are very important in our clinical and non-clinical practice and they may really improve research and education.

In conclusion, there are currently many studies on AI in EM focusing on the improvement of patient’ care. Most of them reported positive results, while in other cases studies are too preliminary rather than non-conclusive. Indeed, AI is a very promising tool to improve both medical and non-medical activities in the field of EM and different options are now available. Nevertheless, since most of the studies are very small rather than pilot, a clear discussion among EM physicians is now necessary in order to better define the application of this technology in the real world by maximizing benefits and reducing risks. Chatbots based on generative AI are surely the most used tools by EM doctors and students but they still show many problems and limitations, including the lack of control over information sources.

## Data Availability

Personal data are still unpublished. Othere data come from the general literature and are still available.
